# Hypothyroxinaemia during gestation is associated with low ferritin and increased levels of inflammatory markers

**DOI:** 10.1530/ETJ-23-0163

**Published:** 2024-03-04

**Authors:** Victor J M Pop, Johannes G Krabbe, Maarten Broeren, Wilmar Wiersinga, Margaret P Rayman

**Affiliations:** 1 Department of Medical Psychology, Tilburg University, The Netherlands; 2 Department of Clinical Chemistry and Laboratory Medicine, Medisch Spectrum Twente, Medlon BV, Enschede, The Netherlands; 3 Department of Clinical Chemistry, Maxima Medical Centre, Veldhoven, The Netherlands; 4 Department of Endocrinology and Metabolism, Academic Medical Center, University of Amsterdam, The Netherlands; 5 Department of Nutritional Sciences, Faculty of Health and Medical Sciences, University of Surrey, Guildford, UK

**Keywords:** inflammation, isolated hypothyroxinaemia, pregnancy

## Abstract

**Objective:**

Pregnancy is a state of physiological inflammation facilitating implantation. Early isolated hypothyroxinaemia (IH) and increased inflammation (including obesity) have been associated with severe obstetric complications. The current study evaluated the association between IH, low ferritin and inflammation parameters (interleukin 6 (IL-6), C-reactive protein (CRP), human chorionic gonadotrophin (hCG) and obesity. Moreover, the course of these parameters throughout pregnancy was evaluated in relation to IH.

**Methods:**

In the cross-sectional study (A) at 12 weeks, 2759 women participated and 2433 participated in the longitudinal study (B) with assessments at 12, 20 and 28 weeks gestation. At the first trimester, 122 (4.4%) IH women (free thyroxine (FT4) <5th percentile, normal TSH levels) were compared with 2114 (76.6%) reference women (FT4 between tenth and 90th percentiles, normal thyrotrophin (TSH) levels), in study B these figures were 99 (4.1%) and 1847 (75.9%), respectively.

**Results:**

Cross-sectionally, compared to reference women, IH was independently associated with low ferritin (<5th percentile, OR: 2.6, 95% CI: 1.4–4.9), high CRP (>95th percentile: OR: 1.9, 95% CI: 1.04–3.7), low hCG (<median, OR: 2.1, 95% CI: 1.40–3.16), obesity (BMI > 30, OR: 1.7, 95% CI: 1.12.9) and higher age (OR: 1.1, 95% CI: 1.04–1.15). Longitudinally, compared to reference women, women with IH at 12 weeks gestation showed persistently and significantly lower ferritin and hCG levels, and persistently higher CRP and IL-6 levels throughout gestation.

**Conclusion:**

Gestational IH could be viewed as a condition of increased inflammation, as reported in non-thyroidal illness syndrome. Less favourable inflammation parameters and low iron status during early gestation in IH women seem to persist throughout gestation.

## Introduction

In recent decades, a new concept has been defined with regard to thyroid hormone (TH) function during early pregnancy: isolated hypothyroxinaemia (IH) (1, 2). This refers to the condition of women with low free thyroxin (FT4) concentration (<2.5th or 5th percentiles) with thyrotrophin (TSH) within normal reference interval (2.5–97.5th percentiles) (1, 2). IH has repeatedly been associated with the ‘big four’ obstetric complications: pre-term birth (PTB), intra-uterine growth retardation, pre-eclampsia and diabetes gravidarum as well as impaired offspring neurodevelopment (2). A very recent meta-analysis confirmed these associations, but they were only observed when using lower FT4 thresholds, 2.5th or fifth percentile, but not with the tenth percentile (3, 4). However, the same meta-analysis included four studies in which 895 women were treated with TH and showed no risk mitigation compared to 1247 control women (3). The uncertainty about treatment is still there resulting in different strategies between the American and European Thyroid Associations (4). It is thus all the more surprising that – apart from the effect of iodine intake on IH – there are very few reports on possible other determinants of IH during (early) gestation.

Iron is important for thyroid function in the form of thyroid peroxidase (TPO), a haeme-dependent enzyme with iron at its active centre; it iodinates tyrosine residues within thyroglobulin to produce TH (5). Hence, in iodine-sufficient countries, first trimester TH hypofunction is significantly associated with low iron status (6). The WHO estimates that 38% of pregnant women suffer from anaemia worldwide and that 50% of these cases are due to iron deficiency making it an important health issue during pregnancy (7). Serum or plasma ferritin concentrations normally reflect body iron stores (8). A significant negative correlation between ferritin and TSH levels has been observed, while there is a positive correlation between ferritin and FT4 (6). Although the relationship between iron deficiency and hypothyroidism has repeatedly been demonstrated, its association with IH has hardly been investigated.

An important alternative source modulating FT4 concentrations is the human chorionic gonadotropin hormone (hCG); hCG binds to the TSH receptor thereby increasing TH secretion during early gestation (9), but its possible association with IH is not known. hCG also has an important immune modulatory function during (early) pregnancy. Conception and implantation are characterized by a state of physiological inflammation accompanied by a heightened response of T-helper cells (Th1 and Th2) resulting in the production of inflammatory cytokines including IL-6 and IL-8 (10). hCG is important for keeping the Th1/Th2 ratio balanced and an increase of the Th1/Th2 ratio (e.g., by low levels of hCG) can result in an elevated production of Th1-mediated cytokines (such as IL-6) increasing the risk of pregnancy complications (10, 11).

C-reactive protein (CRP) is synthesized in the liver and serves as an early marker of inflammation that can activate the immune system (12).

An important methodological issue, when looking at the characteristics of women with IH, is the definition of a reference group. Within this concept it makes little sense to define IH with a FT4 cut-off <2.5th or <5th percentiles and to regard women with, for example, a third or sixth percentile FT4 level, respectively, as belonging to the normal group. Thus, we feel that the reference group should refer to women with neither too low nor too high FT4 concentrations, for example, between the tenth and 90th percentiles. It is reasonable to accept that these 80% of all women reflect adequate levels of FT4.

Therefore, the current project had two major study outcomes. First, at a cross-sectional level, we aimed to evaluate at first trimester, the possible association between low ferritin (as a proxy for iron depletion), hCG, different inflammation parameters (IL-6, CRP) and IH. Second, at a longitudinal level, we aimed to evaluate the possible changes of these biological parameters throughout pregnancy in women with IH.

## Materials and methods

### Procedure and participants

The current study is part of the Brabant Study project (BrSt) the design of which has been described in detail elsewhere (13). In sum, between June 2018 and December 2022, eligible pregnant women in the South-East Brabant area were invited to participate by their community midwife at first antenatal control. Exclusion criteria were age <18, the existence of twin pregnancy, pregnancy after assisted reproductive technology and drug intake interfering with thyroid function (such as lithium, amiodarone). Overall, of the eligible 4480 women who became pregnant during this recruitment period, the number of women who gave written informed consent for participation was 2865 (63%). The BrSt was completed in accordance with the Declaration of Helsinki as revised in 2013 and approved by the Medical Ethical Committee of the Máxima Medical Hospital, Veldhoven (No. NL6409.015.17).

**Figure 1:**
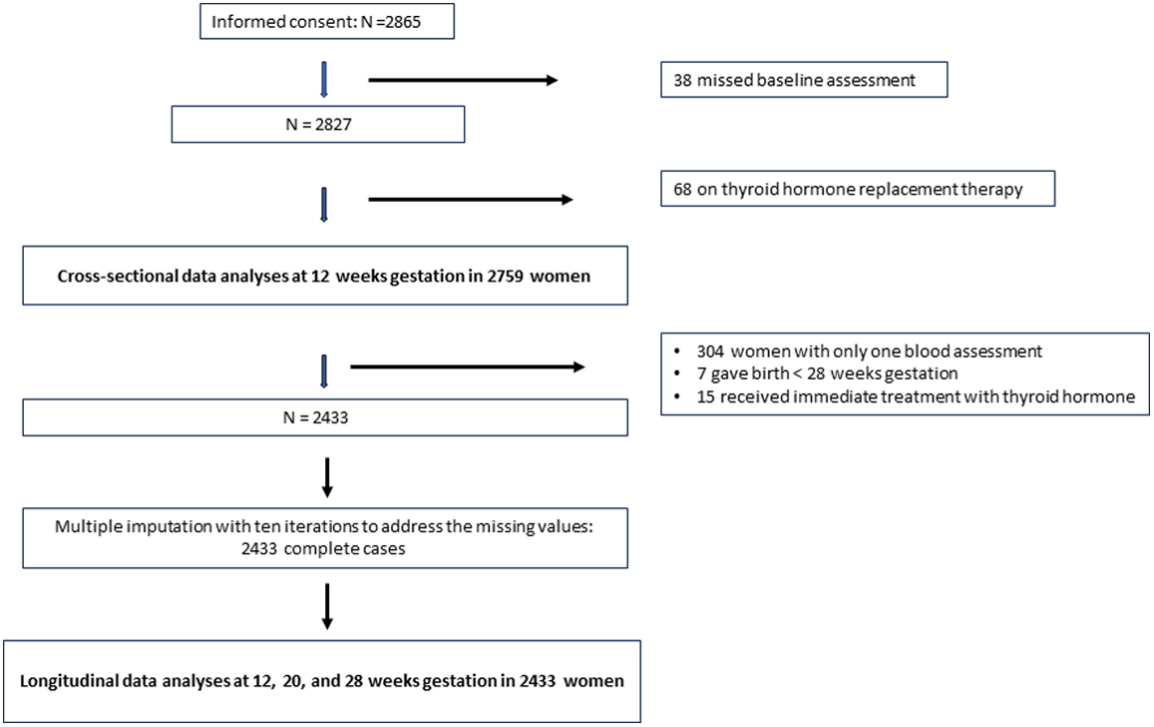
Flow chart of pregnant women of the Brabant Study who were included in the cross-sectional and the longitudinal follow-up study.

The sample used for cross-sectional analyses consisted of 2759 women (flow chart of Fig. 1): 38 women did not complete the baseline questionnaires and 68 women were on thyroxine replacement therapy.

In the sample used for longitudinal analyses, several women missed blood assessments and the women with only one assessment were excluded (Fig. 1). Then, missing values were imputed in women with at least two blood assessments by means of multiple imputation (see Statistical analysis section) resulting in a final longitudinal sample of 2433 women (flow chart Fig. 1).

### Assessments

At baseline (12 weeks gestation), the women completed a set of questionnaires including demographic, obstetric and lifestyle aspects.

### Biological assessments in Li-heparin plasma at 12, 20 and 28 weeks gestation

All assessments were performed on the Cobas® e601 platform of Roche Diagnostics.

#### Thyroid function

TSH, FT4 and TPO-Ab concentrations were determined using electrochemiluminescence immunoassays. Within-laboratory coefficients of variation were: 2,4% at 0.5 mIU/L and 2.9% at 36.8 mIU/L for TSH, 2.6% at 14 pmol/L and 3.3% at 68 pmol/L for FT4, and 6.3% at 52 kU/L and 4.3% at 130 kU/L for anti-TPO. A TPO-Ab titer >35 kU/L was defined as TPO-Ab positive. We defined the reference ranges of TSH and FT4 during pregnancy in TPO-Ab negative women excluding women on thyroid hormone medication, using the 2.5th and 97.5th percentile to define the lower and upper limit of normal thyroid function. IH was defined as an FT4 concentration <5th percentile with normal TSH (2.5th–97.5th percentile). The reference group consisted of women with FT4 between tenth and 90th percentiles with TSH within reference limits.

#### Human chorionic gonadotropin

Human chorionic gonadotrophin (hCG) was assessed by an electrochemiluminescence immunoassay. The within-laboratory coefficients of variation was 5.6% at a concentration of 360 IU/L. This HCG assay detects the holo-hormone, ‘nicked’ forms of hCG, the β-core fragment and the free β-subunit.

#### Ferritin

Ferritin was measured using an electrochemiluminescence assay. The lower limit of quantification was 1 µg/L. Within-laboratory coefficients of variation were 1.8% at 74 µg/L and 2.1% at 360 µg/L. Values above 100 µg/L were rounded to the nearest multiple of ten. Because several studies reported a significant difference of ferritin level according to parity, we defined a low ferritin level (reflecting low iron status) below the (parity specific) fifth percentile cut-off (14).

#### C-reactive protein

CRP (not high sensitive CRP) was measured using an immunoturbidimetric assay. The lower limit of quantification was 0.6 mg/L. Within-laboratory coefficients of variation were 2.2% at 7.3 mg/L and 1.9% at 52 mg/L. We defined an elevated CRP level using the 95th percentile cut-off.

#### Interleukin 6

IL-6 was measured using an electrochemiluminescence immunoassay. The lower limit of quantification was 1.5 ng/L. Within-laboratory coefficients of variation were 2.3% at 39 ng/L and 1.8% at 250 ng/L. We defined an elevated IL-6 level using the 95th percentile cut-off.

### Statistical analysis

Statistical analyses were performed using the IBM SPSS Statistics for Windows v 28.0 (IBM Corp). At a cross-sectional level, descriptive statistics was used to analyse possible differences between IH women and the reference group (chi-square tests for categorical variables, *t*-test for parametric in case of normal distribution (age, BMI) and Mann–Whitney *U* test (M-W-U) for non-parametric tests in the case of non-normal distribution (CRP, IL-6, ferritin and hCG). The M-W-U test compares medians between two groups and evaluates whether the ranks of the scores for the two groups differ significantly. This difference is expressed in a Z score. Subsequently, we performed unadjusted (single) and adjusted (multivariate) logistic regressions (OR, 95% CI) analyses with IH at 12 weeks as dependent variable and low ferritin, low hCG, high CRP and high IL-6 as independent dichotomous and age as continuous variables.

At a longitudinal level, there were women who missed blood assessments at different trimesters. We performed a Little’s Missing Completely at Random (MCAR) test showing a *P* for all variables from 0.15 to 0.82 indicating that the missing values were completely at random (15). Then, the missing values were imputed in women with at least two blood assessments by means of multiple imputation (16). Auto-correlation function plots revealed convergence of ten imputed datasets (16). In this imputed longitudinal dataset, we finally used GLM-ANOVA to compare changes of mean concentrations of FT4, (log)TSH, (log)ferritin, (log)CRP,(log) hCG, and (log)IL-6 throughout pregnancy between women with IH and those of the reference group at 12 weeks gestation.

## Results

### Cross-sectional data

The characteristics of the cross-sectional sample of 2759 women are shown in Table 1.

**Table 1:** Characteristics of pregnant women with assessment of FT4, TSH, TPO-Ab, hCG, ferritin and CRP at 12 weeks gestation (cross-sectional sample: *n* = 2759) and with assessments at 12, 20 and 28 weeks gestation (imputed longitudinal sample: *n* = 2433). Data are presented as *n* (%), mean ± s.d. or as median (range).

	Cross-sectional sample	Longitudinal sample
Demographic features		
Age, years	31.3 ± 3.6	31.4 ± 3.6
Higher education level^a^	1861 (67.4)	1682 (69.1)
With partner	2662 (96.7)	2313 (95.0)
Paid job	2657 (96.2)	2323 (95.5)
Lifestyle characteristics		
Smoking	47 (1.7)	35 (1.4)
Alcohol intake	19 (0.7)	15 (0.6)
BMI	24.3 ± 4.3	24.2 ± 4.3
Obstetric features		
Parity		
Primipara	1375 (49.8)	1228 (50.5)
Multipara	1387 (50.2)	1205 (49.5)
Previous miscarriage	768 (27.8)	650 (26.7)
Different sub-categories^b^		
Hypothyroxinaemia	122 (4.4)	99 (4.1)
Reference group	2114 (76.6)	1847 (75.9)
Overt unknown hypothyroidism	11 (0.5)	Excluded
Overt hyperthyroidism	33 (1.2)	26 (1.1)
Sub-clinical hypothyroidism		
TSH >3.37 and <10 mIU/L	99 (3.6)	81 (3.3)
TSH >10 mIU/L	4 (0.1)	Excluded
Sub-clinical hyperthyroidism	31 (1.1)	25 (1.0)
TPO-Ab >35 IU/L	218 (7.9)	178 (7.3)
Low ferritin	147 (5.3)	120 (4.9)
High CRP	138 (5.0)	125 (5.1)
High IL-6	136 (4.9)	123 (5.1)
Low hCG (<median)	1374 (49.8)	1221 (50.2)
Biological parameters		
TSH mIU/L	1.21 (0.01–63.5)	1.20 (0.01–9.3)
FT4 pmol/L	14.6 (5.6–29.5)	14.7 (9.2–29.4)
hCG IU/L	62000 (6400–443418)	63000 (6300–282,282)
Ferritin µg/L	59 (5–536)	59 (5–536)
CRP mg/L	3.3 (0.6–119)	3.2 (0.6–119)
IL-6 ng/L	1.5 (1.5–35.6)	1.5 (1.5–34.4)

^a^Higher education: bachelor degree or higher;

^b^Hypothyroxinaemia: FT4 < 5th percentile (<11.90 pmol/L), TSH within 2.5–97.5th percentiles. Reference group: FT4 between 10th and 90th percentiles (12.50–17.01 pmol/L), TSH within 2.5–97.5th percentiles. Overt hypothyroidism: FT4 < 2.5th (<11.50 pmol/L) and TSH > 97.5th percentile (>3.37 mIU/L), TPO-Ab– women. Overt hyperthyroidism: FT4 > 97.5th (>18.87 pmol/L) and TSH1 < 2.5th percentile (0.13 mIU/L), TPO-Ab– women. Sub-clinical hypothyroidism: FT4 within 2.5–97.5th percentiles, TSH > 97.5th percentile. Sub-clinical hyperthyroidism: FT4 within 2.5–97.5th percentiles, TSH < 2.5th percentile. Low ferritin: <5th percentile: in primiparous women: <23 µg/L; in multiparous women: <14 µg/L. High CRP: >95th percentile: > 14.58 µg/L. High IL-6: >95th percentile: > 3.71 ng/L. Low hCG: < median, < 63,000 IU/L.

There were 122 women with IH (FT4 <11.90 pmol/L, TSH within reference range, 0.13–3.33 mIU/L) and 2114 (76.5%) belonged to the reference group (FT4 between 12.50 and 17.01 pmol/L, TSH within reference range). We defined low hCG using the median as cut-off which was <63.000 IU/L (Table 1). There were 222 (9.9%) obese women with a BMI >30 (WHO criteria).

In Table 2, the characteristics of the IH group are compared with that of the reference group.

**Table 2:** Characteristics at 12 weeks gestation of 122 women with IH (FT4 <5th percentile with TSH within 2.5th–95.5th percentiles) compared to the reference group (*n* = 2114) women with adequate FT4 levels (between tenth and 90th percentiles with TSH within 2.5–97.5th percentiles).

	Women with IH	Reference group	*P*		
			*χ*^2^	T	M-W-U
Demographic features					
Age, years	32.4 ± 3.8	31.2 ± 3.6		<0.001	
Higher education leve^l*^	85 (69.7)	1505 (71.2)	0.72		
With partner	116 (95)	2012 (95.2)	0.96		
Paid job	118 (96.7)	2038 (96.4)	0.85		
Lifestyle characteristics					
Smoking	3 (2.5)	37 (1.8)	0.56		
Alcohol intake	1 (0.8)	12 (0.6)	0.72		
BMI	25.9 ± 5.3	24.2 ± 4.2		<0.001	
Obstetric features					
Parity					
Primipara	57 (46.7)	1045 (49.4)	0.56		
Multipara	65 (53.3)	1069 (50.6)			
Previous miscarriage	38 (31.3)	551 (26.1)	0.21		
Biological parameters					
FT4, pmol/L	11.2± 0.5	14.6 ± 1.13		<0.001	
TPO-Ab >35 IU/L	8 (6.6)	143 (6.7)	0.99		
TSH mIU/L	1.44 (0.40–2.45)	1.29 (0.15–3.33)			0.040
hCG IU/L	51814 (10,408–37,724)	62311 (7200–238,510)			<0.001
Ferritin µg/L	51 (5–311)	60 (5–521)			0.006
CRP mg/L	5.5 (0.12–71)	3.3 (0.6–85)			<0.001
IL-6 ng/L	1.5 (1.5–15.3)	1.5 (1.5–35.7)			0.003

IH, isolated hypothyroxinaemia: FT4 <5th percentile (<11.90 pmol/L), TSH within 2.5–97.5th percentiles of TPO-Ab negative women; Reference group: adequate FT4 levels, between tenth and 90th percentile (12.50–17.01 pmol/L), TSH within 2.5th–97.5th percentiles of TPO-Ab negative women.

^*^bachelor degree or higher.

M-W-U: Mann–Whitney U test.

**Figure 2:**
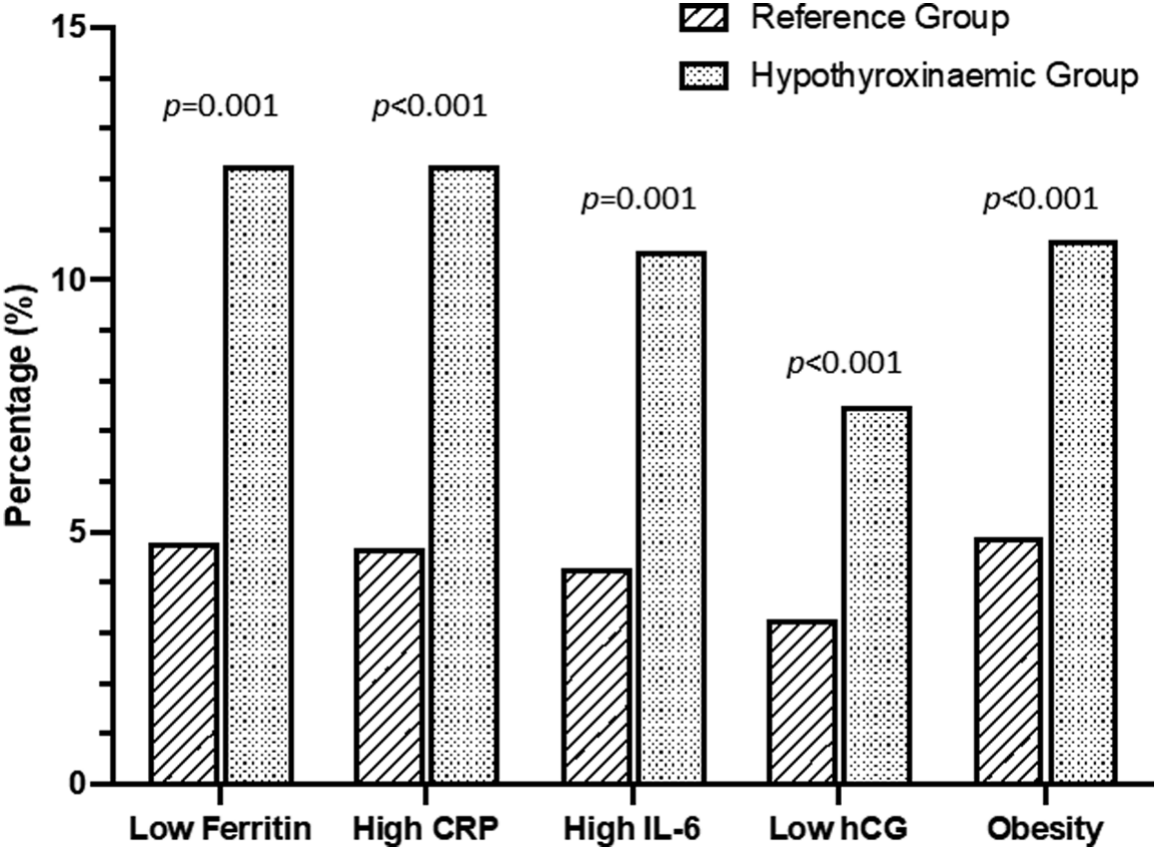
Number of cases (%) with low ferritin (<5th percentile), high CRP and high IL-6 (>95th percentile), low hCG (below median) and high BMI (>30) of 122 women with isolated hypothyroxinaemia (FT4 <5th percentile with TSH between 2.5th and 97.5th percentiles) compared with the number of cases of the 2114 women of the reference group (FT4 between tenth and 90th percentile with TSH between 2.5th and 97.5th percentile) at 12 weeks gestation. *P* refers to chi-square tests: *df* = 1.

The IH women were significantly older and had a significantly higher mean BMI. Also, there was a (highly) significant difference in hCG, CRP, IL-6 and ferritin levels between the two groups. In Fig. 2, the percentage of women with low ferritin, high CRP, IL-6, low hCG and obesity (BMI >30) between both groups are compared. There were significantly more women with low ferritin in the IH group (12.3%) than in the reference group (4.8%): *χ* ^2^ (1) = 10.7,* P* = 0.001.

Similarly, there were significantly more women with high CRP level in the IH group (12.3%) than in the reference group (4.7%), (*χ*^2^ (1) = 13.5, *P* < 0.001), and more IH women (10.6%) than reference women (4.3%) with high IL-6 levels, (*χ*^2^ (1) = 10.5, *P* = 0.001). In the IH group, there were significantly more obese women (BMI >30), (*χ*^2^ (1) = 13.6, *P* < 0.001) and significantly more women with low hCG (< the median), (*χ*^2^ (1) = 18.8, *P* < 0.001).

In Table 3, unadjusted and adjusted logistic regressions are shown, performed in 2236 women (122 IH and 2114 women of the reference group) with IH as dependent variable. Single logistic regression showed that logTSH, low ferritin status, high CRP, high IL-6, obesity as well as low hCG concentrations and increasing age were all significantly associated with IH (Table 3).

**Table 3:** Univariate and multivariate logistic regression analyses at 12 weeks gestation in 2236 women (122 with IH and 2114 women with adequate FT4 levels); dependent variable: IH, independent variables: low ferritin, high CRP, IL-6, low hCG, logTSH, obesity and age.

	OR (95 % CI)	*P*
Univariate		
Low ferritin	2.6 (1.4–4.9)	0.002
High CRP	2.8 (1.6–5.0)	<0.001
High IL-6	2.4 (1.3–4.6)	0.006
Obesity	2.4 (1.5–3.8)	<0.001
Higher age	1.1 (1.04–1.15)	<0.001
Low hCG	2.4 (1.58–3.51)	<0.001
logTSH	2.5 (1.09–5.79)	0.035
Multivariate		
Low ferritin	2.6 (1.4–4.9)	0.002
High CRP	1.9 (1.04–3.7)	0.037
High IL-6	1.8 (0.9–3.6)	0.092
Obesity	1.7 (1.1–2.9)	0.033
Higher age	1.1 (1.04–1.15)	<0.001
Low hCG	2.1 (1.40–3.16)	<0.001
logTSH	2.1 (0.88–4.80)	0.11

See legend of Table 1 for definition of sub-categories. Obesity: BMI >30 (WHO).

At a multivariate level (Table 3) most of these associations (except IL-6 and TSH) with IH persisted; low ferritin status and low hCG independently increased the likelihood of IH 2.6 times and 2.1 times, respectively, while a high CRP status and obesity increased the likelihood by a factor of 2.1 and 1.7, respectively. Every year of increasing age augmented the likelihood of IH by 10%.

### Longitudinal data

**Figure 3:**
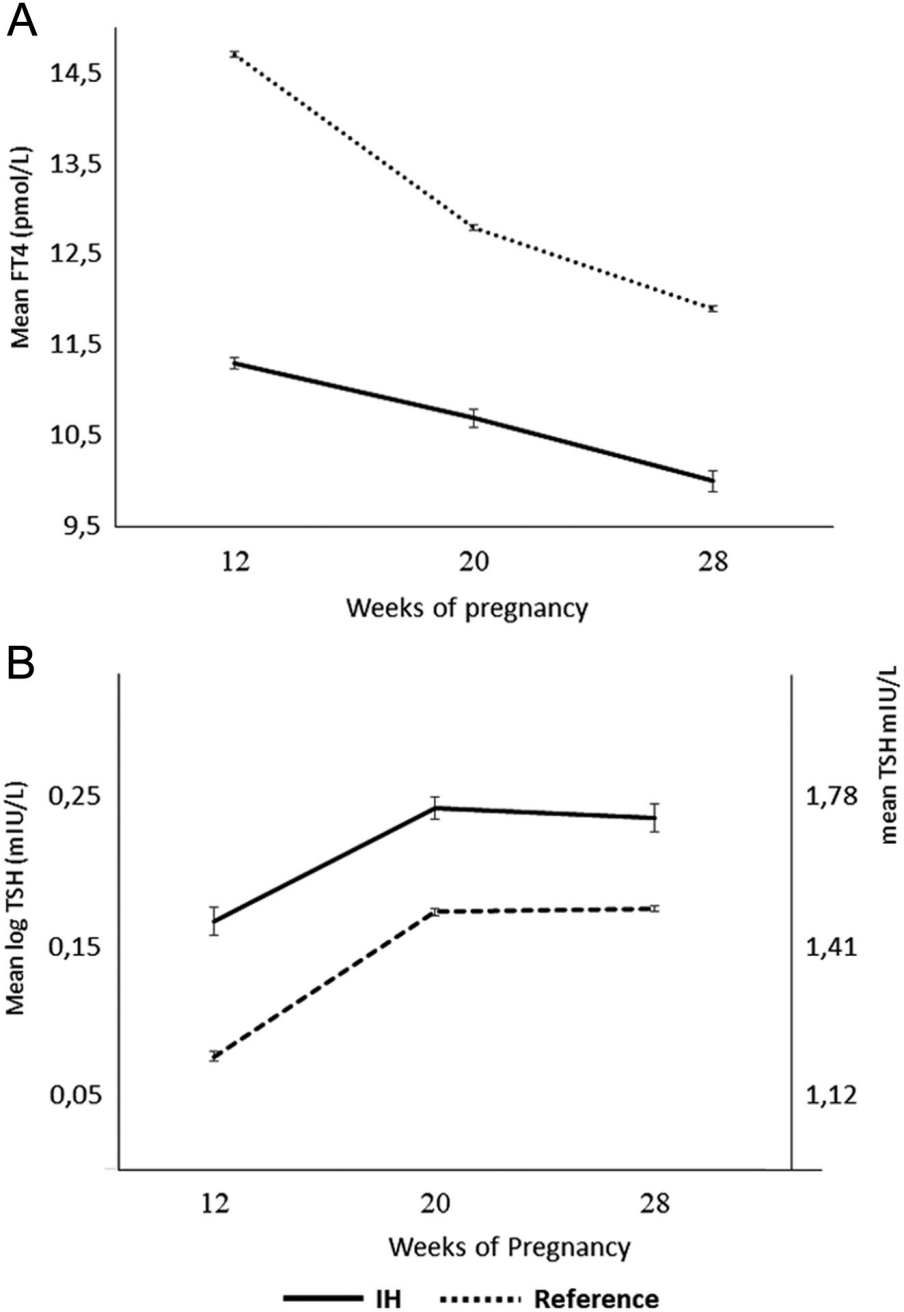
Mean concentrations of FT4 (A) and mean log(TSH) (B) throughout gestation of 99 women with IH (isolated hypothyroxinaemia) compared to 1847 women with adequate FT4 levels (between tenth and 90th percentiles) at 12 weeks gestation.

The characteristics of the longitudinal cohort of 2433 women with three biological assessments at 12, 20 and 28 weeks gestation were similar to the original cross-sectional cohort (Table 1). In this cohort of 2433 women, at 12 weeks gestation, there were 99 (4.1%) women with IH and 1847 (75.9%) belonged to the reference group. When we compared the FT4 changes throughout gestation, both groups showed a declining pattern but women with IH at 12 weeks gestation had persistently and significantly lower mean FT4 concentrations, compared to women of the reference group at 12 weeks gestation (Fig. 3A), *F* (1): 564,* P* < 0.001 (GLM-ANOVA).

Also, women with IH had persistently higher mean (log)TSH levels throughout gestation (Fig. 3B: *F* (1): 6.7, *P* = 0.009).

**Figure 4:**
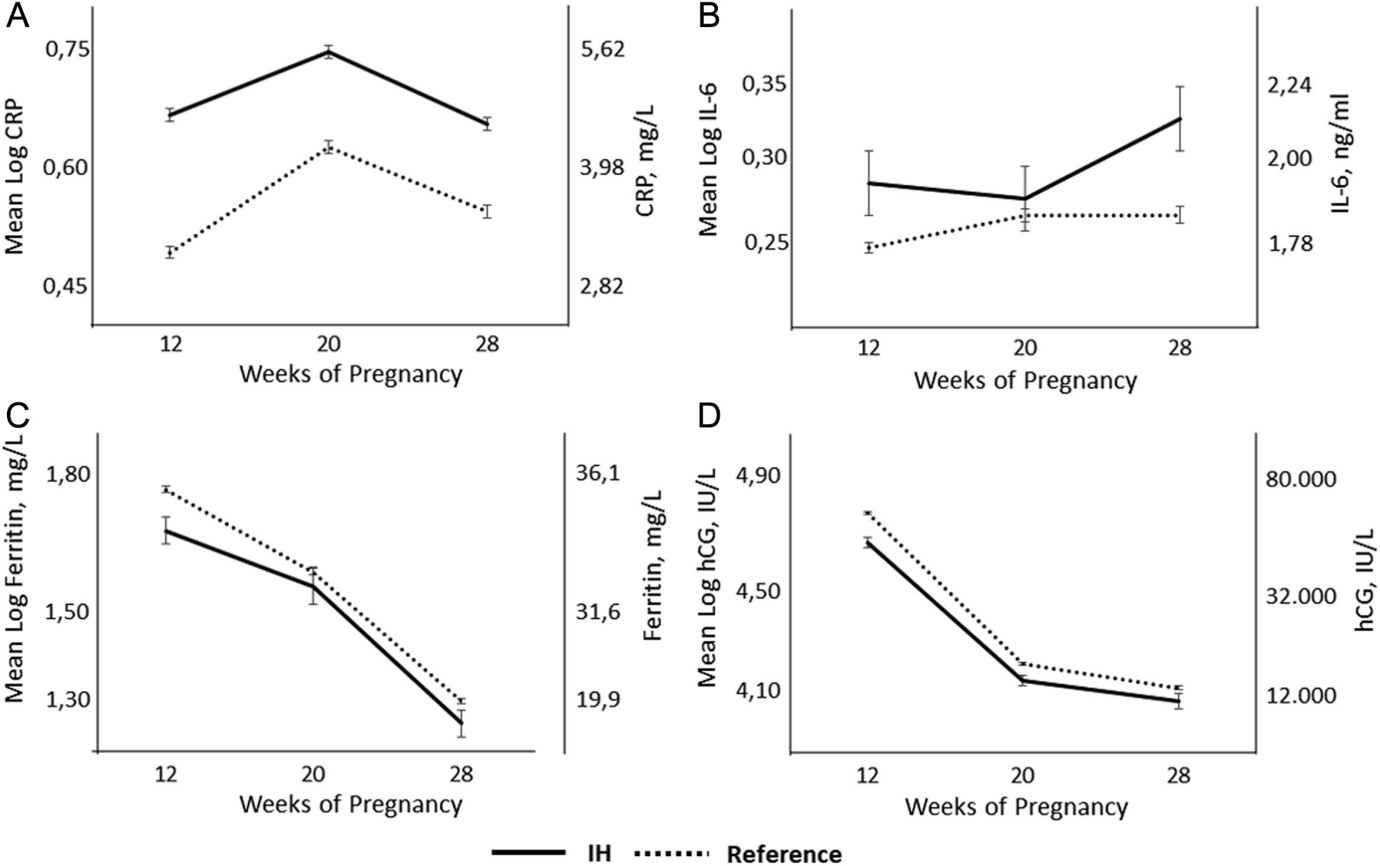
Comparison between 99 IH women and 1847 women with adequate FT4 levels (FT4 between tenth and 90th percentiles) at 12 weeks gestation with regard to: mean (log)CRP, (A); mean (log)IL-6, (B); mean (log)fer, (C); mean (log)hCG, (D), throughout gestation. The left *Y*-axis shows mean log concentrations, the right *Y*-axis shows the corresponding linear concentrations. IH: isolated hypothyroxinaemia.

In Fig. 4, the mean concentrations of (log)ferritin, (log)CRP, (log)IL-6 and (log)hCG throughout pregnancy are shown in the 99 women with IH at 12 weeks gestation compared to the 1847 reference women.

Throughout gestation, compared to the reference women, women with IH at 12 weeks gestation had persistently and significantly higher mean (log)CRP, *F* (1): 14.8, *P* < 0.001 (Fig. 4A), and mean logIL-6, *F* = 12.5, *P* < 0.001 (Fig. 4B); they showed persistently and significantly lower mean (log)ferritin, *F* (1): 4.8, *P* = 0.034 (Fig. 4C), and mean (log)hCG, *F* (1) = 12.8, *P* < 0.001 (Fig. 4D) throughout gestation.

## Discussion

### Cross-sectional analyses at 12 weeks

The current study shows that first trimester low plasma ferritin levels (as a proxy for low iron status), low hCG, high CRP, high IL-6 levels, higher age and obesity (BMI >30) were independently associated with IH.

### Representativeness of current birth cohort

The current sample is representative of the total obstetric population in The Netherlands in terms of parity, mean age of pregnant women and previous abortion figures. (National Data on Dutch Birth Outcome, Perined) (17). But the women in our cohort are predominantly white Caucasian, almost all of them had a partner, up to two out of three were highly educated and the number of women who smoked or drank alcohol and who had a BMI >30 was lower than in the general Dutch population (CBS data 2021) (18). The response rate of 63% after one single invitation by the community midwife is relatively high for population based research and comparable to that of a similar birth cohort study in The Netherlands, the Generation-R study (61%) (19). Previous six birth cohorts of pregnant women, recruited during the past 30 years of the same area, showed a response rate of 76–82% (20). The lower response rate of the current study is mainly to be explained by the COVID pandemic that occurred during the recruitment period. Women were less willing to participate because of the fear of possible COVID infection during an (additional) blood assessment. The high number of highly educated women in the current study is less likely to be a consequence of selection bias; this area of south-east Netherlands was nominated as being the smartest in the world in 2011 by the international think-tank Intelligent Community Forum (ICF) in New York.

### Possible mechanisms to explain findings

Several mechanisms might explain the current findings. First, the haeme-dependent enzyme, TPO, is required for TH synthesis, so low ferritin levels, as a proxy for iron deficiency, will decrease TPO activity and may therefore contribute to impaired TH synthesis (6). Hypothyroidism may result in inadequate protection to cells resulting in increased oxidative stress (OS) (21, 22), which in turn has been closely related to inflammation markers such as IL-6 and CRP (21). Second, inflammation (high IL-6) may suppress the deiodination of T4 by deiodinases D1 and D2 eventually resulting in lower TH in tissue (23). Third, because hCG activates the TSH receptor, lower hCG levels will result in lower FT4 and this might explain why in the current study, IH women showed lower hCG concentrations (9). Besides, hCG also has an immune modulatory function and low hCG levels will enhance inflammation by production of Il-6 further impairing TH synthesis (10).

Obesity has a significant inflammatory component and overweight individuals have increased serum levels of inflammatory cytokines such as tumor necrosis factor alpha (TNF-α), CRP and interleukins (IL-6, IL-18) (24). In the non-pregnant state, obesity is associated with a chronic, low-grade inflammatory state, termed ‘meta-inflammation’ or metabolically induced inflammation (24). High levels of inflammation (including obesity) – often resulting in high concentrations of pro-inflammatory parameters – have repeatedly been associated with obstetric complications including: preterm birth, pre-eclampsia, gestational diabetes, intra-uterine growth retardation or large for gestational age babies (25). The independent association between obesity, IH and inflammation in the current study is intriguing; it is well known that low TH concentrations will result in a decline of metabolic processes. Excessive weight gain is one of the key symptoms of (often unknown) overt hypothyroidism. Are women with high BMI, a chronic low-grade inflammatory state, before they become pregnant especially at risk for IH during pregnancy reflecting in fact increased inflammation?

The independent association between age and IH is interesting. ‘Inflammageing’ in general describes a sterile, non-resolving, low-grade and chronic inflammation that progressively increases with increasing age (26). Age-related cell senescence can give rise to significant pro-inflammatory signaling (including the production of IL-6) and highlight the complex interplay between general cell senescence, immunosenescence and inflammation (26). Although pregnant women (by definition) are relatively young, increasing age in the fertile period has repeatedly been associated with obstetric complications including pre-term birth (27). The finding that, in the adjusted model, IL-6 was no longer significantly (*P* = 0.09) related to IH can easily be explained by the important confounding effect of obesity on inflammation; at 12 weeks gestation, obese women were 3.5 times more likely to have high IL-6 levels (OR: 3.49, 95% CI: 2.3–5.2, *P* < 0.001) attenuating the independent effect of IL-6 on IH.

### Isolated hyperthyroxinaemia: a non-thyroidal illness syndrome?

In fact, despite the lack of information on FT3, TT3 and rT3 in the current study, a challenging hypothesis to explain the association between inflammation parameters and IH could be that low FT4 (with normal TSH) in IH could be regarded as an example of a (mild) non-thyroidal illness syndrome (NTIS). One of the key features of NTIS is the absence of increased serum TSH in the face of decreased serum T3 and T4, pointing to altered set-point regulation of the hypothalamus–pituitary–thyroid axis (28). Interestingly, this key feature is incorporated in the definition of IH. Already back in 1990, the Escobar group in Madrid described thyroid hormone economy in pregnant rats as a ‘physiological animal model of NTIS’ (29). They suggested that the decrease of TH pools leading to lower levels of TH in most tissues without the elevation of TSH (attenuation of the negative feedback loop) is the normal physiological response to situations where preservation of energy is warranted, as is the case in pregnancy (29). Another study from Amsterdam in pregnant women in an iodine replete area showed that with increasing term, plasma FT4 and FT3 concentrations decreased with a concomitant increase of free rT3 levels, changes that are also found in the NTIS (30). The authors also suggested a physiological adaptation enabling energy conservation during the high metabolic demands of pregnancy (30). Taking these considerations together, is it possible that the physiological state of inflammation in pregnancy to facilitate placentation in some cases is not adequately counterbalanced by anti-oxidants, reflected by low hCG levels and resulting in elevated levels of IL-6 and CRP, especially in women with low iron status and/or obesity and at higher age?

### Longitudinal analyses throughout gestation

The longitudinal analyses of the current showed that women with IH and the reference women during early gestation followed the ‘classical pattern’ of FT4 and TSH changes during gestation: a gradually decrease of FT4 towards end gestation ‘mirrored’ by a gradual increase of TSH (2). However, IH women did not ‘recover’ from their initial low TH status during the first trimester: they persistently showed lower mean FT4 levels and higher mean (log)TSH levels compared to the reference group towards the end of gestation. Similarly, women with initially low iron stores (as reflected by low serum ferritin) were more likely to have persistently lower iron stores throughout the pregnancy compared to the reference group. Also, women with IH in the first trimester were more likely to have persistently less favourable inflammation parameter (low hCG, high CRP and high IL-6) outcomes compared to those with adequate FT4 levels.

### Strengths and limitations

The current study has strengths and limitations. A major strength is the relatively large sample size, especially with regard to the longitudinal design. We did not assess FT3 levels; it would be challenging to compare the FT3 status of hypothyroxinaemic women with those with adequate FT4 levels. There is still some debate concerning the accuracy of FT4 assessment during (especially the second half of) gestation due to the alterations in binding proteins (31). Therefore, several guidelines suggest using total T4 (TT4) as an alternative measure for TH function during pregnancy (31). However, a recent study showed that FT4 and not TT4 levels were significantly associated with adverse pregnancy and child outcome suggesting that FT4 is a better representation of TH availability (32). Although iodine status – the most important determinant of IH – was not assessed, previous research in the same area showed that at a population level there is substantial evidence for adequate iodine intake in pregnancy: the number of neonates with increased TSH levels (>5 mIU/L) at congenital heel screening was 1.5%, which is <3%, a generally accepted cut-off showing sufficient iodine intake at a population level (33, 34). Ethnicity, lifestyle and demographic features were not representative of the total population in the Netherlands which hampers the generalizability of the study.

### Possible implications at research and clinical level

At a research level, findings of the current study relating IH to increased inflammation suggest that IH seems not to be simply an endocrinological issue. Especially the independent association between IH and obesity (a low-grade chronic state of inflammation), between IH and low ferritin as a proxy for anaemia (both conditions that are highly prevalent in pregnant women), and between IH and inflammation parameters underlines the possible multifactorial origin of IH. There is an urgent need for further research assessing TH parameters that are specifically related to NTIS (FT3 and rT3 levels) explaining whether IH in fact should be regarded as an epiphenomenon of increased inflammation rather than an endocrinological condition (28). Also, there is an urgent need for RCTs where pregnant women with IH should be treated with a combination of TH, iron supplementation and anti-oxidants rather than with TH alone as suggested by others (35).

Finally, we are left with the most important clinical question: should we treat IH in daily practice? On the one hand, IH is unequivocally associated with poor obstetric outcome, while on the other hand there is growing evidence now that substitution with TH does not benefit obstetric outcome and infant development (3, 4). Interestingly, the possible benefit of T4 substitution in NTIS is also still under debate (28). Increased inflammation, low iron status, high BMI, higher age as well as IH are all associated with major obstetric complications (2, 3, 36). So far, the best option in clinical practice seems that women with IH should be strictly monitored for being at risk for obstetric complications due to the possible increased inflammatory status associated with IH. An CRP assessment in women with IH could be performed (cheap, user-friendly) followed by adequate monitoring of those with elevated CRP levels: there is substantial literature showing an association between high CRP levels and obstetric complications (like PTB) (37).

In conclusion, the current study suggests that IH during early gestation is associated with persistently lower FT4 levels throughout gestation. IH could be regarded as a phenomenon of low iron status and increased inflammation, a status which persists during pregnancy. The NTIS could be a challenging model to explain these associations.

## Declaration of interest

The authors declare that there is no conflict of interest that could be perceived as prejudicing the impartiality of the study reported.

## Funding

V J M Pop received funding of Tilburg University by an appointment of a PhD student on the project and payment of the laboratory assessments. V J M Pop received funding from Rochehttp://dx.doi.org/10.13039/100004337 Diagnostics Global who delivered the kits for free. M Broeren has no funding to report. J G Krabbe has no funding to report. W Wiersinga has no funding to report. M P Rayman has no funding to report.

## Author contribution statement

V J M Pop designed the Brabant Study, performed the analyses and conceptualized the first draft of the manuscript (lead). V J M Pop, W Wiersinga and M P Rayman equally reviewed and edited the final version of the manuscript. M Broeren was responsible for the blood sample analyses (lead) and edited and reviewed the laboratory section of the method section (supporting) and critically reviewed the manuscript. J G Krabbe critically revised the manuscript and supported the writing and editing of the manuscript.
